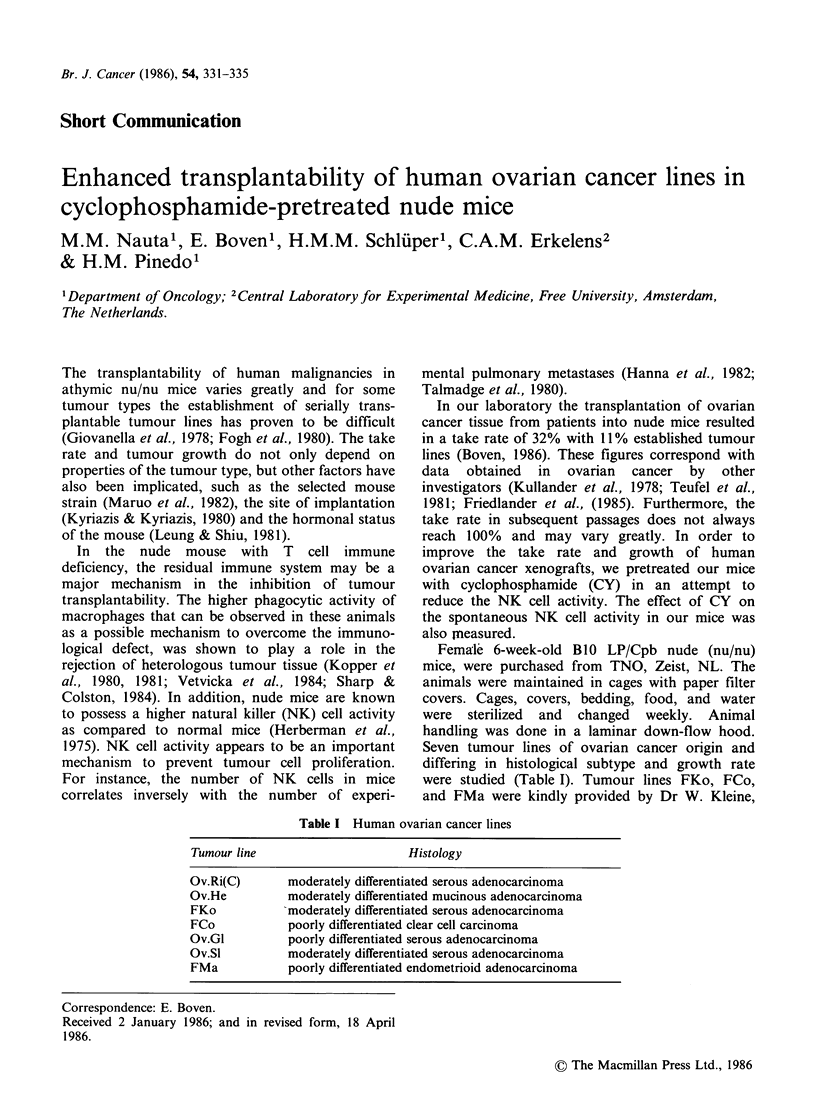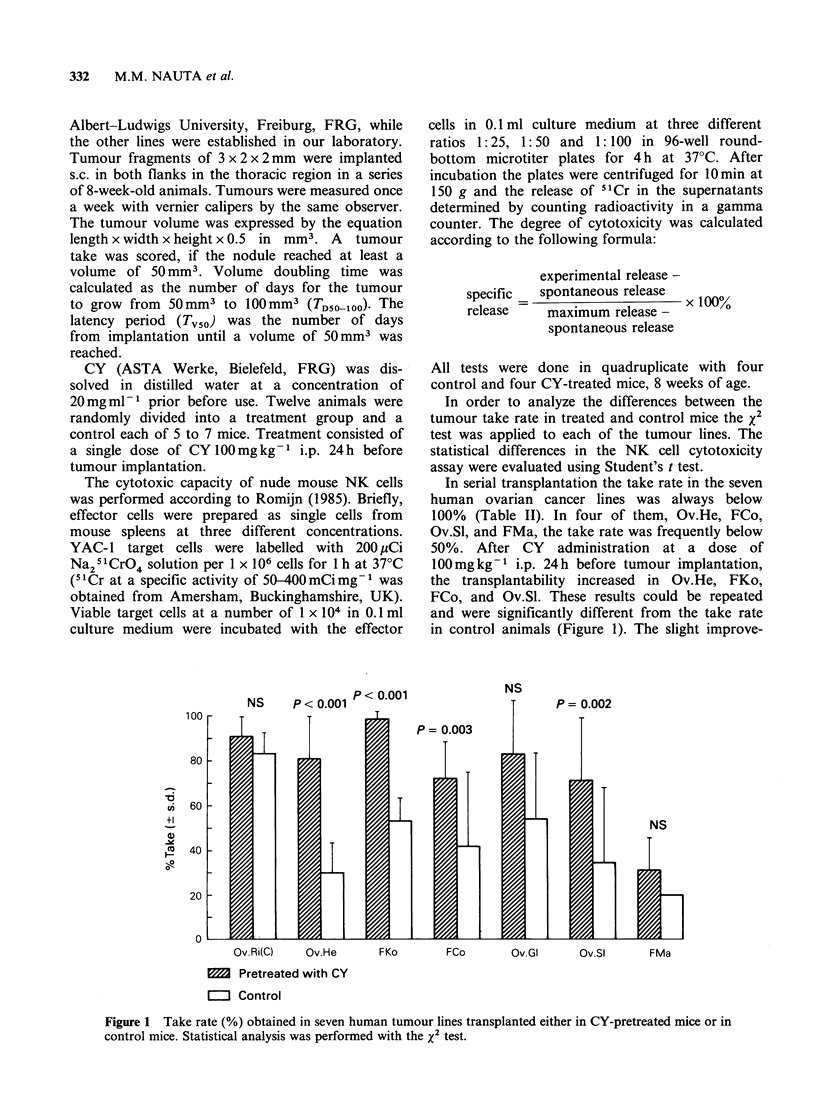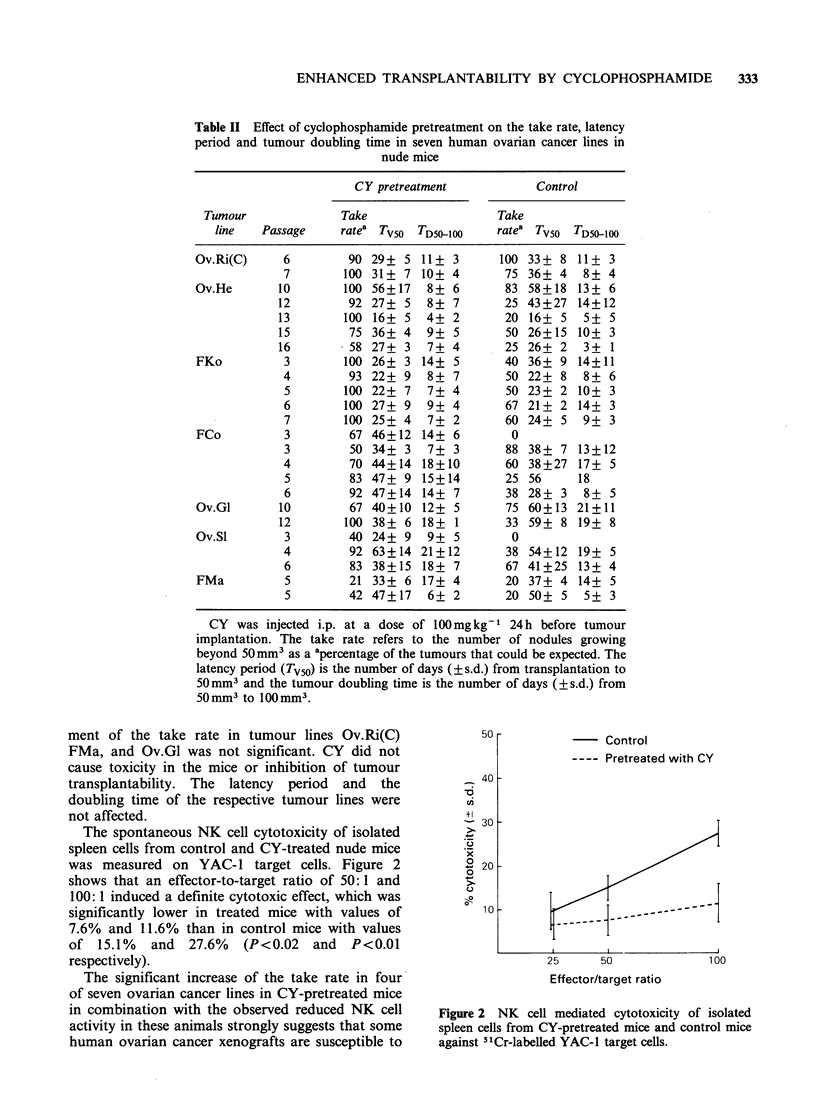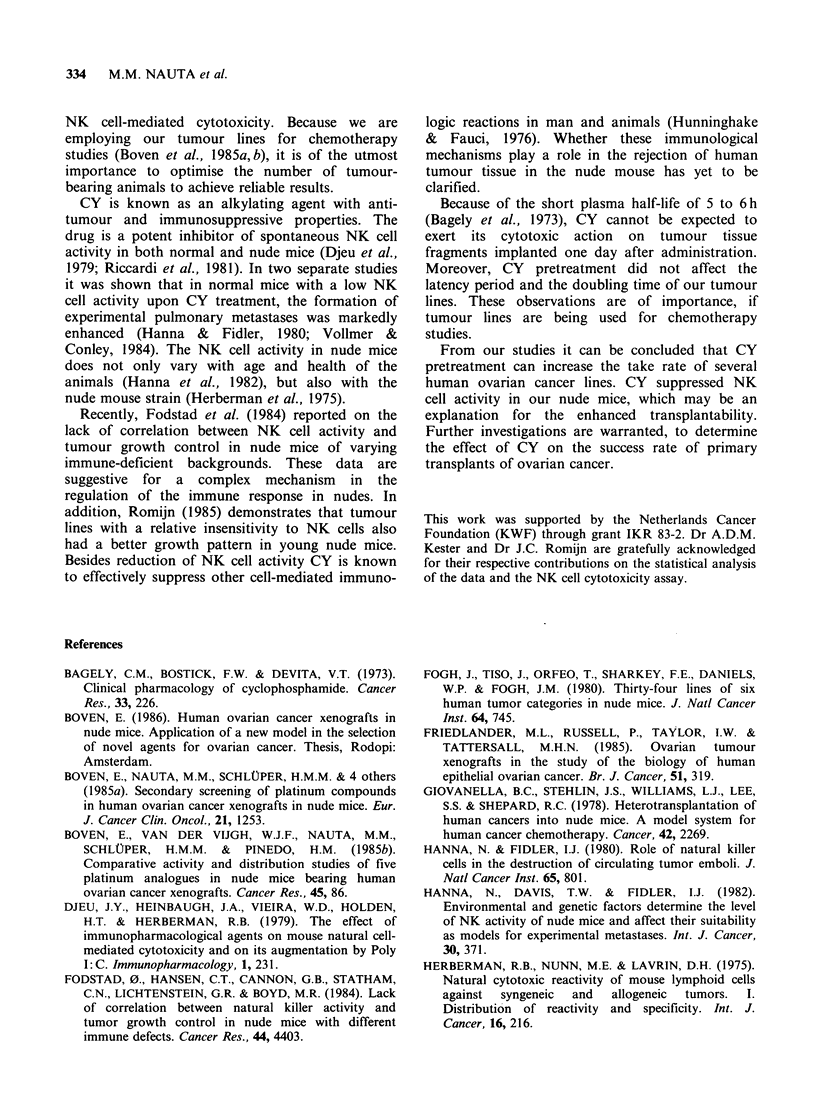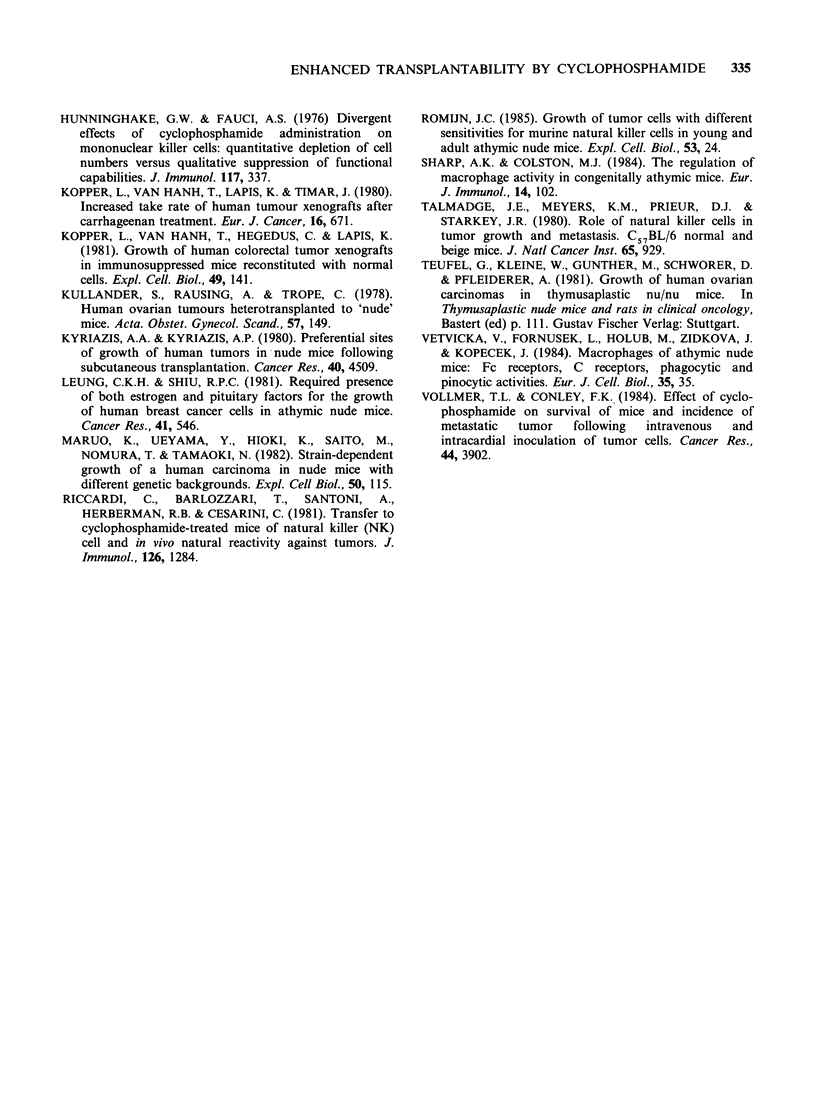# Enhanced transplantability of human ovarian cancer lines in cyclophosphamide-pretreated nude mice.

**DOI:** 10.1038/bjc.1986.181

**Published:** 1986-08

**Authors:** M. M. Nauta, E. Boven, H. M. Schlüper, C. A. Erkelens, H. M. Pinedo


*Br. J. Cancer* (1986), **54**, 331–335

### Short Communication

### Enhanced transplantability of human ovarian cancer lines in cyclophosphamide-pretreated nude mice

M.M. Nauta[1], E. Boven[1], H.M.M. Schlüper[1], C.A.M. Erkelens[2] & H.M. Pinedo[1]

[1]*Department of Oncology;* [2]*Central Laboratory for Experimental Medicine, Free University, Amsterdam, The Netherlands.*

The transplantability of human malignancies in athymic nu/nu mice varies greatly and for some tumour types the establishment of serially transplantable tumour lines has proven to be difficult (Giovanella *et al.*, 1978; Fogh *et al.*, 1980). The take rate and tumour growth do not only depend on properties of the tumour type, but other factors have also been implicated, such as the selected mouse strain (Maruo *et al.*, 1982), the site of implantation (Kyriazis & Kyriazis, 1980) and the hormonal status of the mouse (Leung & Shiu, 1981).

In the nude mouse with T cell immune deficiency, the residual immune system may be a major mechanism in the inhibition of tumour transplantability. The higher phagocytic activity of macrophages that can be observed in these animals as a possible mechanism to overcome the immunological defect, was shown to play a role in the rejection of heterologous tumour tissue (Kopper *et al.*, 1980, 1981; Vetvicka *et al.*, 1984; Sharp & Colston, 1984). In addition, nude mice are known to possess a higher natural killer (NK) cell activity as compared to normal mice (Herberman *et al.*, 1975). NK cell activity appears to be an important mechanism to prevent tumour cell proliferation. For instance, the number of NK cells in mice correlates inversely with the number of experimental pulmonary metastases (Hanna *et al.*, 1982; Talmadge *et al.*, 1980).

In our laboratory the transplantation of ovarian cancer tissue from patients into nude mice resulted in a take rate of 32% with 11% established tumour lines (Boven, 1986). These figures correspond with data obtained in ovarian cancer by other investigators (Kullander *et al.*, 1978; Teufel *et al.*, 1981; Friedlander *et al.*, (1985). Furthermore, the take rate in subsequent passages does not always reach 100% and may vary greatly. In order to improve the take rate and growth of human ovarian cancer xenografts, we pretreated our mice with cyclophosphamide (CY) in an attempt to reduce the NK cell activity. The effect of CY on the spontaneous NK cell activity in our mice was also measured.

Female 6-week-old B10 LP/Cpb nude (nu/nu) mice, were purchased from TNO, Zeist, NL. The animals were maintained in cages with paper filter covers. Cages, covers, bedding, food, and water were sterilized and changed weekly. Animal handling was done in a laminar down-flow hood. Seven tumour lines of ovarian cancer origin and differing in histological subtype and growth rate were studied (Table I). Tumour lines FKo, FCo, and FMa were kindly provided by Dr W. Kleine,

**Table I** Human ovarian cancer lines

| *Tumour line* | *Histology* |
|---|---|
| Ov.Ri(C) | moderately differentiated serous adenocarcinoma |
| Ov.He | moderately differentiated mucinous adenocarcinoma |
| FKo | moderately differentiated serous adenocarcinoma |
| FCo | poorly differentiated clear cell carcinoma |
| Ov.Gl | poorly differentiated serous adenocarcinoma |
| Ov.Sl | moderately differentiated serous adenocarcinoma |
| FMa | poorly differentiated endometrioid adenocarcinoma |

Correspondence: E. Boven.
Received 2 January 1986; and in revised form, 18 April 1986.

© The Macmillan Press Ltd., 1986

Albert–Ludwigs University, Freiburg, FRG, while the other lines were established in our laboratory. Tumour fragments of 3 × 2 × 2 mm were implanted s.c. in both flanks in the thoracic region in a series of 8-week-old animals. Tumours were measured once a week with vernier calipers by the same observer. The tumour volume was expressed by the equation length × width × height × 0.5 in $mm^3$. A tumour take was scored, if the nodule reached at least a volume of $50\,mm^3$. Volume doubling time was calculated as the number of days for the tumour to grow from $50\,mm^3$ to $100\,mm^3$ ($T_{D50-100}$). The latency period ($T_{V50}$) was the number of days from implantation until a volume of $50\,mm^3$ was reached.

CY (ASTA Werke, Bielefeld, FRG) was dissolved in distilled water at a concentration of $20\,mg\,ml^{-1}$ prior before use. Twelve animals were randomly divided into a treatment group and a control each of 5 to 7 mice. Treatment consisted of a single dose of CY $100\,mg\,kg^{-1}$ i.p. 24 h before tumour implantation.

The cytotoxic capacity of nude mouse NK cells was performed according to Romijn (1985). Briefly, effector cells were prepared as single cells from mouse spleens at three different concentrations. YAC-1 target cells were labelled with $200\,\mu Ci$ $Na_2{}^{51}CrO_4$ solution per $1 \times 10^6$ cells for 1 h at 37°C ($^{51}Cr$ at a specific activity of 50–400 $mCi\,mg^{-1}$ was obtained from Amersham, Buckinghamshire, UK). Viable target cells at a number of $1 \times 10^4$ in 0.1 ml culture medium were incubated with the effector cells in 0.1 ml culture medium at three different ratios 1:25, 1:50 and 1:100 in 96-well round-bottom microtiter plates for 4 h at 37°C. After incubation the plates were centrifuged for 10 min at 150 *g* and the release of $^{51}Cr$ in the supernatants determined by counting radioactivity in a gamma counter. The degree of cytotoxicity was calculated according to the following formula:

$$\text{specific release} = \frac{\text{experimental release} - \text{spontaneous release}}{\text{maximum release} - \text{spontaneous release}} \times 100\%$$

All tests were done in quadruplicate with four control and four CY-treated mice, 8 weeks of age.

In order to analyze the differences between the tumour take rate in treated and control mice the $\chi^2$ test was applied to each of the tumour lines. The statistical differences in the NK cell cytotoxicity assay were evaluated using Student's *t* test.

In serial transplantation the take rate in the seven human ovarian cancer lines was always below 100% (Table II). In four of them, Ov.He, FCo, Ov.Sl, and FMa, the take rate was frequently below 50%. After CY administration at a dose of $100\,mg\,kg^{-1}$ i.p. 24 h before tumour implantation, the transplantability increased in Ov.He, FKo, FCo, and Ov.Sl. These results could be repeated and were significantly different from the take rate in control animals (Figure 1). The slight improve-

**Figure 1** Take rate (%) obtained in seven human tumour lines transplanted either in CY-pretreated mice or in control mice. Statistical analysis was performed with the $\chi^2$ test.

**Table II** Effect of cyclophosphamide pretreatment on the take rate, latency period and tumour doubling time in seven human ovarian cancer lines in nude mice

| | | *CY pretreatment* | | | *Control* | | |
|---|---|---|---|---|---|---|---|
| *Tumour line* | *Passage* | *Take rate*[a] | $T_{V50}$ | $T_{D50-100}$ | *Take rate*[a] | $T_{V50}$ | $T_{D50-100}$ |
| Ov.Ri(C) | 6 | 90 | 29± 5 | 11± 3 | 100 | 33± 8 | 11± 3 |
| | 7 | 100 | 31± 7 | 10± 4 | 75 | 36± 4 | 8± 4 |
| Ov.He | 10 | 100 | 56±17 | 8± 6 | 83 | 58±18 | 13± 6 |
| | 12 | 92 | 27± 5 | 8± 7 | 25 | 43±27 | 14±12 |
| | 13 | 100 | 16± 5 | 4± 2 | 20 | 16± 5 | 5± 5 |
| | 15 | 75 | 36± 4 | 9± 5 | 50 | 26±15 | 10± 3 |
| | 16 | 58 | 27± 3 | 7± 4 | 25 | 26± 2 | 3± 1 |
| FKo | 3 | 100 | 26± 3 | 14± 5 | 40 | 36± 9 | 14±11 |
| | 4 | 93 | 22± 9 | 8± 7 | 50 | 22± 8 | 8± 6 |
| | 5 | 100 | 22± 7 | 7± 4 | 50 | 23± 2 | 10± 3 |
| | 6 | 100 | 27± 9 | 9± 4 | 67 | 21± 2 | 14± 3 |
| | 7 | 100 | 25± 4 | 7± 2 | 60 | 24± 5 | 9± 3 |
| FCo | 3 | 67 | 46±12 | 14± 6 | 0 | | |
| | 3 | 50 | 34± 3 | 7± 3 | 88 | 38± 7 | 13±12 |
| | 4 | 70 | 44±14 | 18±10 | 60 | 38±27 | 17± 5 |
| | 5 | 83 | 47± 9 | 15±14 | 25 | 56 | 18 |
| | 6 | 92 | 47±14 | 14± 7 | 38 | 28± 3 | 8± 5 |
| Ov.Gl | 10 | 67 | 40±10 | 12± 5 | 75 | 60±13 | 21±11 |
| | 12 | 100 | 38± 6 | 18± 1 | 33 | 59± 8 | 19± 8 |
| Ov.Sl | 3 | 40 | 24± 9 | 9± 5 | 0 | | |
| | 4 | 92 | 63±14 | 21±12 | 38 | 54±12 | 19± 5 |
| | 6 | 83 | 38±15 | 18± 7 | 67 | 41±25 | 13± 4 |
| FMa | 5 | 21 | 33± 6 | 17± 4 | 20 | 37± 4 | 14± 5 |
| | 5 | 42 | 47±17 | 6± 2 | 20 | 50± 5 | 5± 3 |

CY was injected i.p. at a dose of $100\,mg\,kg^{-1}$ 24 h before tumour implantation. The take rate refers to the number of nodules growing beyond $50\,mm^3$ as a [a]percentage of the tumours that could be expected. The latency period ($T_{V50}$) is the number of days (±s.d.) from transplantation to $50\,mm^3$ and the tumour doubling time is the number of days (±s.d.) from $50\,mm^3$ to $100\,mm^3$.

ment of the take rate in tumour lines Ov.Ri(C) FMa, and Ov.Gl was not significant. CY did not cause toxicity in the mice or inhibition of tumour transplantability. The latency period and the doubling time of the respective tumour lines were not affected.

The spontaneous NK cell cytotoxicity of isolated spleen cells from control and CY-treated nude mice was measured on YAC-1 target cells. Figure 2 shows that an effector-to-target ratio of 50:1 and 100:1 induced a definite cytotoxic effect, which was significantly lower in treated mice with values of 7.6% and 11.6% than in control mice with values of 15.1% and 27.6% ($P<0.02$ and $P<0.01$ respectively).

The significant increase of the take rate in four of seven ovarian cancer lines in CY-pretreated mice in combination with the observed reduced NK cell activity in these animals strongly suggests that some human ovarian cancer xenografts are susceptible to

**Figure 2** NK cell mediated cytotoxicity of isolated spleen cells from CY-pretreated mice and control mice against $^{51}Cr$-labelled YAC-1 target cells.

NK cell-mediated cytotoxicity. Because we are employing our tumour lines for chemotherapy studies (Boven *et al.*, 1985*a*, *b*), it is of the utmost importance to optimise the number of tumour-bearing animals to achieve reliable results.

CY is known as an alkylating agent with anti-tumour and immunosuppressive properties. The drug is a potent inhibitor of spontaneous NK cell activity in both normal and nude mice (Djeu *et al.*, 1979; Riccardi *et al.*, 1981). In two separate studies it was shown that in normal mice with a low NK cell activity upon CY treatment, the formation of experimental pulmonary metastases was markedly enhanced (Hanna & Fidler, 1980; Vollmer & Conley, 1984). The NK cell activity in nude mice does not only vary with age and health of the animals (Hanna *et al.*, 1982), but also with the nude mouse strain (Herberman *et al.*, 1975).

Recently, Fodstad *et al.* (1984) reported on the lack of correlation between NK cell activity and tumour growth control in nude mice of varying immune-deficient backgrounds. These data are suggestive for a complex mechanism in the regulation of the immune response in nudes. In addition, Romijn (1985) demonstrates that tumour lines with a relative insensitivity to NK cells also had a better growth pattern in young nude mice. Besides reduction of NK cell activity CY is known to effectively suppress other cell-mediated immunologic reactions in man and animals (Hunninghake & Fauci, 1976). Whether these immunological mechanisms play a role in the rejection of human tumour tissue in the nude mouse has yet to be clarified.

Because of the short plasma half-life of 5 to 6 h (Bagely *et al.*, 1973), CY cannot be expected to exert its cytotoxic action on tumour tissue fragments implanted one day after administration. Moreover, CY pretreatment did not affect the latency period and the doubling time of our tumour lines. These observations are of importance, if tumour lines are being used for chemotherapy studies.

From our studies it can be concluded that CY pretreatment can increase the take rate of several human ovarian cancer lines. CY suppressed NK cell activity in our nude mice, which may be an explanation for the enhanced transplantability. Further investigations are warranted, to determine the effect of CY on the success rate of primary transplants of ovarian cancer.

This work was supported by the Netherlands Cancer Foundation (KWF) through grant IKR 83-2. Dr A.D.M. Kester and Dr J.C. Romijn are gratefully acknowledged for their respective contributions on the statistical analysis of the data and the NK cell cytotoxicity assay.

### References

BAGELY, C.M., BOSTICK, F.W. & DEVITA, V.T. (1973). Clinical pharmacology of cyclophosphamide. *Cancer Res.*, **33**, 226.

BOVEN, E. (1986). Human ovarian cancer xenografts in nude mice. Application of a new model in the selection of novel agents for ovarian cancer. Thesis, Rodopi: Amsterdam.

BOVEN, E., NAUTA, M.M., SCHLÜPER, H.M.M. & 4 others (1985*a*). Secondary screening of platinum compounds in human ovarian cancer xenografts in nude mice. *Eur. J. Cancer Clin. Oncol.*, **21**, 1253.

BOVEN, E., VAN DER VIJGH, W.J.F., NAUTA, M.M., SCHLÜPER, H.M.M. & PINEDO, H.M. (1985*b*). Comparative activity and distribution studies of five platinum analogues in nude mice bearing human ovarian cancer xenografts. *Cancer Res.*, **45**, 86.

DJEU, J.Y., HEINBAUGH, J.A., VIEIRA, W.D., HOLDEN, H.T. & HERBERMAN, R.B. (1979). The effect of immunopharmacological agents on mouse natural cell-mediated cytotoxicity and on its augmentation by Poly I:C. *Immunopharmacology*, **1**, 231.

FODSTAD, Ø., HANSEN, C.T., CANNON, G.B., STATHAM, C.N., LICHTENSTEIN, G.R. & BOYD, M.R. (1984). Lack of correlation between natural killer activity and tumor growth control in nude mice with different immune defects. *Cancer Res.*, **44**, 4403.

FOGH, J., TISO, J., ORFEO, T., SHARKEY, F.E., DANIELS, W.P. & FOGH, J.M. (1980). Thirty-four lines of six human tumor categories in nude mice. *J. Natl Cancer Inst.* **64**, 745.

FRIEDLANDER, M.L., RUSSELL, P., TAYLOR, I.W. & TATTERSALL, M.H.N. (1985). Ovarian tumour xenografts in the study of the biology of human epithelial ovarian cancer. *Br. J. Cancer*, **51**, 319.

GIOVANELLA, B.C., STEHLIN, J.S., WILLIAMS, L.J., LEE, S.S. & SHEPARD, R.C. (1978). Heterotransplantation of human cancers into nude mice. A model system for human cancer chemotherapy. *Cancer*, **42**, 2269.

HANNA, N. & FIDLER, I.J. (1980). Role of natural killer cells in the destruction of circulating tumor emboli. *J. Natl Cancer Inst.* **65**, 801.

HANNA, N., DAVIS, T.W. & FIDLER, I.J. (1982). Environmental and genetic factors determine the level of NK activity of nude mice and affect their suitability as models for experimental metastases. *Int. J. Cancer*, **30**, 371.

HERBERMAN, R.B., NUNN, M.E. & LAVRIN, D.H. (1975). Natural cytotoxic reactivity of mouse lymphoid cells against syngeneic and allogeneic tumors. I. Distribution of reactivity and specificity. *Int. J. Cancer*, **16**, 216.

HUNNINGHAKE, G.W. & FAUCI, A.S. (1976) Divergent effects of cyclophosphamide administration on mononuclear killer cells: quantitative depletion of cell numbers versus qualitative suppression of functional capabilities. *J. Immunol.* **117,** 337.

KOPPER, L., VAN HANH, T., LAPIS, K. & TIMAR, J. (1980). Increased take rate of human tumour xenografts after carrhageenan treatment. *Eur. J. Cancer,* **16,** 671.

KOPPER, L., VAN HANH, T., HEGEDUS, C. & LAPIS, K. (1981). Growth of human colorectal tumor xenografts in immunosuppressed mice reconstituted with normal cells. *Expl. Cell. Biol.,* **49,** 141.

KULLANDER, S., RAUSING, A. & TROPE, C. (1978). Human ovarian tumours heterotransplanted to 'nude' mice. *Acta. Obstet. Gynecol. Scand.,* **57,** 149.

KYRIAZIS, A.A. & KYRIAZIS, A.P. (1980). Preferential sites of growth of human tumors in nude mice following subcutaneous transplantation. *Cancer Res.,* **40,** 4509.

LEUNG, C.K.H. & SHIU, R.P.C. (1981). Required presence of both estrogen and pituitary factors for the growth of human breast cancer cells in athymic nude mice. *Cancer Res.,* **41,** 546.

MARUO, K., UEYAMA, Y., HIOKI, K., SAITO, M., NOMURA, T. & TAMAOKI, N. (1982). Strain-dependent growth of a human carcinoma in nude mice with different genetic backgrounds. *Expl. Cell Biol.,* **50,** 115.

RICCARDI, C., BARLOZZARI, T., SANTONI, A., HERBERMAN, R.B. & CESARINI, C. (1981). Transfer to cyclophosphamide-treated mice of natural killer (NK) cell and *in vivo* natural reactivity against tumors. *J. Immunol.,* **126,** 1284.

ROMIJN, J.C. (1985). Growth of tumor cells with different sensitivities for murine natural killer cells in young and adult athymic nude mice. *Expl. Cell. Biol.,* **53,** 24.

SHARP, A.K. & COLSTON, M.J. (1984). The regulation of macrophage activity in congenitally athymic mice. *Eur. J. Immunol.,* **14,** 102.

TALMADGE, J.E., MEYERS, K.M., PRIEUR, D.J. & STARKEY, J.R. (1980). Role of natural killer cells in tumor growth and metastasis. $C_{57}$BL/6 normal and beige mice. *J. Natl Cancer Inst.* **65,** 929.

TEUFEL, G., KLEINE, W., GUNTHER, M., SCHWORER, D. & PFLEIDERER, A. (1981). Growth of human ovarian carcinomas in thymusaplastic nu/nu mice. In *Thymusaplastic nude mice and rats in clinical oncology,* Bastert (ed) p. 111. Gustav Fischer Verlag: Stuttgart.

VETVICKA, V., FORNUSEK, L., HOLUB, M., ZIDKOVA, J. & KOPECEK, J. (1984). Macrophages of athymic nude mice: Fc receptors, C receptors, phagocytic and pinocytic activities. *Eur. J. Cell. Biol.,* **35,** 35.

VOLLMER, T.L. & CONLEY, F.K. (1984). Effect of cyclophosphamide on survival of mice and incidence of metastatic tumor following intravenous and intracardial inoculation of tumor cells. *Cancer Res.,* **44,** 3902.